# A randomised trial of carbetocin versus syntometrine in the management of the third stage of labour

**DOI:** 10.1111/j.1471-0528.2006.01105.x

**Published:** 2006-12

**Authors:** SW Leung, PS Ng, WY Wong, TH Cheung

**Affiliations:** Department of Obstetrics and Gynaecology, The Chinese University of Hong Kong, Prince of Wales Hospital Shatin, New Territories, Hong Kong SAR

**Keywords:** Carbetocin, primary postpartum haemorrhage, syntometrine, third stage of labour, uterotonic agents

## Abstract

**Objective:**

Syntometrine is an effective uterotonic agent used in preventing primary postpartum haemorrhage but has adverse effects including nausea, vomiting, hypertension and coronary artery spasm. Carbetocin is a newly developed long-acting oxytocin analogue that might be used as an uterotonic agent. We compare the efficacy and safety of intramuscular (IM) carbetocin with IM syntometrine in preventing primary postpartum haemorrhage.

**Design:**

Prospective, double-blinded, randomised controlled trial.

**Setting:**

Delivery suite of a university-based obstetrics unit.

**Population:**

Women with singleton pregnancy achieving vaginal delivery after and throughout 34 weeks.

**Methods:**

Three hundred and twenty-nine eligible women were randomised to receive either a single dose of 100 microgram IM carbetocin or 1 ml IM syntometrine (a mixture of 5 iu oxytocin and 0.5 mg ergometrine) at the end of second stage of labour.

**Main outcome measures:**

Difference in haemoglobin drop measured 2 days after delivery between the two groups.

**Results:**

There was no difference in the drop of haemoglobin concentration within the first 48 hours between the two groups. The incidence of additional oxytocic injections, postpartum haemorrhage (blood loss ≥ 500 ml) and retained placenta were also similar. The use of carbetocin was associated with significant lower incidence of nausea (relative risk [RR] 0.18, 95% confidence interval [CI] 0.04–0.78), vomiting (RR 0.1, 95% CI 0.01–0.74), hypertension 30 minutes (0 versus 8 cases, *P* < 0.01) and 60 minutes (0 versus 6 cases, *P* < 0.05) after delivery but a higher incidence of maternal tachycardia (RR 1.68, 95% CI 1.03–3.57).

**Conclusions:**

IM carbetocin is as effective as IM syntometrine in preventing primary postpartum haemorrhage after vaginal delivery. It is less likely to induce hypertension and has a low incidence of adverse effect. It should be considered as a good alternative to conventional uterotonic agents used in managing the third stage of labour.

## Introduction

Primary postpartum haemorrhage resulting from uterine atony is a major cause of maternal morbidity and mortality.^1^,^2^ Various prophylactic strategies have been used to prevent this potential life-threatening emergency. Systematic reviews have concluded that active management of third stage of labour, particularly the prophylactic use of uterotonic agents can significantly decrease the incidence of postpartum haemorrhage compared with that of expectant management.^3^–^6^ An ideal uterotonic agent should promote prompt, strong and sustained uterine contractions after intramuscular (IM) injection without any significant adverse effects.

Syntometrine is a mixture of oxytocin and ergotamine, and 1 ml of syntometrine contains 5 iu oxytocin and 0.5 mg ergometrine. This mixture combines the rapid onset of action of oxytocin and the sustained effect of ergometrine and is one of the most popular uterotonic agents used in the third stage of labour. IM syntometrine is shown to be as effective as intravenous oxytocin,^7^ and it appears to be associated with a small but statistically significant reduction in the risk of postpartum haemorrhage when compared with oxytocin if blood loss is between 500 and 1000 ml.^5^ Because ergotamine can stimulate smooth muscle contraction and vasoconstriction, it may raise blood pressure^8^ and rarely lead to coronary artery spasm;^9^ therefore, syntometrine is contraindicated in women with asthma, hypertension or cardiac disease.

Carbetocin was first described in 1987^10^ and is a long-acting synthetic analogue of oxytocin, with agonist action. It has a half-life of 40 minutes, and uterine contractions occur in less than 2 minutes after IM or intravenous administration.^11^ The bioavailability is 80% after IM injection,^11^ and the optimal dosage used in the third stage of labour is 100 microgram.^12^ A single intravenous dose of carbetocin has been shown to be as effective as a 16-hour intravenous oxytocin infusion to increase uterine tone and reduce the intraoperative blood loss in women undergoing elective caesarean section.^13^ Another study comparing the uterotonic effect of 100 microgram intravenous carbetocin with 5 unit intravenous oxytocin followed by 20 units intravenous oxytocin infusion in preventing postpartum haemorrhage in women undergoing caesarean section showed a significantly lower incidence of additional oxytocic intervention in carbetocin group compared with that of oxytocin group.^14^ Prophylactic use of carbetocin as uterotonic agent after vaginal delivery among women at increased risk of postpartum haemorrhage has been published in 2004. Women given IM carbetocin required significantly less additional uterotonic intervention than those women who had intravenous oxytocin infusion.^15^ Carbetocin is well tolerated and the safety profile is similar to that of oxytocin.^13^,^14^ These data suggest that IM carbetocin may be a good alternative to IM syntometrine that is commonly used to prevent postpartum haemorrhage. We therefore conducted this randomised trial to compare the efficacy and safety of IM carbetocin with IM syntometrine in managing the third stage of labour among women without any high risk factors for postpartum haemorrhage.

## Methods

This was a prospective, double-blinded, randomised study conducted from July 2004 to March 2005 in the delivery suite of a university hospital in Hong Kong. Women with a singleton pregnancy achieving vaginal delivery beyond 34-week gestation were eligible for the study. Exclusion criteria included the presence of contraindications for the use of either syntometrine or carbetocin that include pre-existing hypertension, pre-eclampsia, asthma, cardiac, renal or liver diseases. Women with high risk factors for primary postpartum haemorrhage such as grand multiparity or presence of uterine fibroids and who required prophylactic oxytocin infusion after delivery were excluded.

Written informed consent was obtained from eligible women before induction or at early stage of labour by the staff of the research team during the morning of each working day from 8.00 hours to 12.00 hours. Women were randomised to receive either 1 ml carbetocin (containing 100 microgram carbetocin) or 1 ml syntometrine (containing 5 units of syntocinon and 0.5 mg ergometrine) given as IM injection at the end of second stage of labour. This was performed by opening a sealed, consecutively numbered, opaque envelope that contained a computer-generated code prepared before the recruitment.

Once an eligible woman with informed consent was about to deliver vaginally, an independent midwife would open up the envelope and draw up the study drug. Either IM carbetocin or syntometrine would be given by this independent midwife upon delivery of the anterior shoulder of the baby and she would then leave the delivery room. The women, the midwives and doctors attending the delivery were blinded to the type of medication injected. Oxytocin infusion, if in progress, was stopped. The third stage of labour was managed as usual by clamping and cutting of umbilical cord, waiting for signs of placental separation and delivering the placenta by controlled cord traction. Additional doses of IM syntometrine or an intravenous oxytocin infusion was prescribed by the attending midwife or doctor if uterine atony was suspected or diagnosed.

The primary outcome variable of this study was the drop in haemoglobin level documented by comparing the maternal haemoglobin concentration on admission to labour ward with that measured 48 hours after delivery. Other secondary outcome variables assessed included the visually estimated amount of blood loss during delivery, the incidence of primary postpartum haemorrhage (defined as blood loss more than 500 ml) and blood transfusion. Maternal blood pressure, pulse and temperature were checked immediately after delivery and repeated 30 and 60 minutes later. The duration of third stage, the incidences of prolonged third stage (duration longer than 30 minutes), the need for manual removal of placenta or additional oxytocic were recorded. We recorded the occurrence of nausea, vomiting, flushing, headache, shivering and pain over injection site by an interview conducted 1 hour after delivery.

Our previous study showed that the mean and standard deviation of postpartum haemoglobin concentration change measured 48 hours after delivery among women treated with syntometrine was 1.34 g/dl and 1.26 g/dl, respectively.^16^ A trial with a power of 90% to detect a difference of 0.5 g/dl in the haemoglobin concentration change with an alpha of 0.05 would require a sample size of 150 in each arm.

All statistical analyses were performed using Statistical Package for Social Science for Windows version 11.0 (SPSS Inc., IL, USA). Differences between the carbetocin and syntometrine groups were compared using the chi-square test or Fisher’s exact test for categorical data and Student’s *t* test for continuous data. Where appropriate, relative risk (RR) and 95% confidence intervals (CI) were calculated. *P* value of less than 0.05 (*P* < 0.05) was considered as statistically significant.

The study protocol was approved by the Clinical Research Ethics Committee of the Faculty of Medicine of the Chinese University of Hong Kong. All women were required to give their written informed consent at the time of recruitment.

## Results

**Figure 1:**
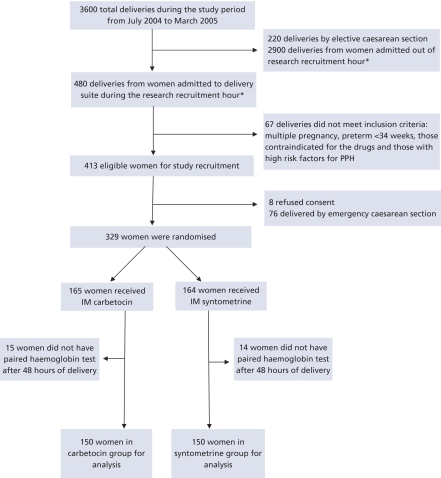
A flow chart of inclusion, exclusion and randomisation of women. PPH, postpartum haemorrhage; *8.00 to 12.00 hours of each working day.

The flow chart of inclusion, exclusion and randomisation of women is shown in Figure 1. There were a total of 3600 deliveries in our hospital during the study period, and 480 deliveries occurred in women admitted to the delivery suite during the research recruitment hour of each morning. A total of 329 women were finally recruited into the study. Fifteen women in the carbetocin group and 14 women in the syntometrine group failed to have a paired haemoglobin test to measure the change in haemoglobin 48 hours after delivery either because they had requested early home or refused further blood taking. These 29 women were excluded, and we therefore had 150 women each in the carbetocin and syntometrine arm in the analysis. There was no significant difference between the two groups in their demographic characteristics (Table 1).

**Table 1:** Demographic characteristics of the study population

Characteristics	Carbetocin (*n* = 150)	Syntometrine (*n* = 150)
Age (years)*	28.3 (5.1)	28.6 (5.6)
Gestation at delivery (weeks)**	39.2 (1.6)	39.4 (1.4)
Nulliparous**	91 (60.7)	92 (61.3)
Previous postpartum haemorrhage**	0 (0)	1 (0.7)
Previous manual removal of placenta**	1 (0.7)	0 (0)
Spontaneous onset of labour**	90 (60)	101 (67.3)
Induction of labour**	60 (40)	49 (32.7)
Augmentation with syntocinon**	28 (18.7)	30 (20)
Epidural analgesia**	30 (20)	32 (21.3)
Normal vaginal delivery**	114 (76)	113 (75.3)
Vacuum extraction**	30 (20)	32 (21.3)
Forceps delivery**	6 (4)	5 (3.3)
Episiotomy**	141 (94)	136 (90.7)
Genital tract trauma**	12 (8)	18 (12)
Birth weight (g)*	3204 (443)	3225 (407)

There is no significant difference between the two groups.

* Data are presented as mean (SD).

** Data are presented as *n* (%).

The mean drop of haemoglobin concentration after delivery was 1.4 g/dl in carbetocin group and 1.5 g/dl in syntometrine group, and the difference was not significant. The haemoglobin concentration dropped >10% in 50 and 54.7% of women and dropped >20% in 16 and 22% of women in the carbetocin and syntometrine group, respectively (Table 2).

**Table 2:** Peripartum haemoglobin concentration

	Carbetocin (*n* = 150)	Syntometrine (*n* = 150)	Mean difference	RR	95% CI
Haemoglobin at onset of labour (g/dl)*	11.6 (1.1)	11.8 (1.2)	−0.2	—	−0.5 to 0
Haemoglobin on postpartum day 2 (g/dl)*	10.2 (1.4)	10.4 (1.5)	−0.2	—	−0.5 to 0.2
Mean fall in haemoglobin (g/dl)*	1.4 (1.1)	1.5 (1.3)	−0.1	—	−0.4 to 0.2
Mean percent drop in haemoglobin concentration*	11.6 (9.4)	12.2 (10.3)	−0.6	—	−2.8 to 1.7
Percent drop of haemoglobin**					
>20%	24 (16)	33 (22)	—	0.72	0.3–1.21
>10%	75 (50)	82 (54.7)	—	0.91	0.77–1.90

* Data are presented as mean (SD).

** Data are presented as *n* (%).

There was no significant difference in the amount of estimated blood loss and the incidence of primary postpartum haemorrhage (≥500 ml) or severe primary postpartum haemorrhage (≥1000 ml) in both groups. One nulliparous woman in the syntometrine group developed massive postpartum haemorrhage of 2 l after vacuum extraction because of uterine atony and retained cotyledon. All cases of primary postpartum haemorrhage occurred either immediately or within 1 hour postdelivery while the woman was still in the delivery room. The incidence of blood transfusion, additional oxytocic injection, prolonged third stage (≥30 minutes) and manual removal of placenta were similar between the two groups (Table 3).

**Table 3:** Secondary outcomes

	Carbetocin (*n* = 150)	Syntometrine (*n* = 150)	Mean difference	RR	95% CI
Mean blood loss (ml)*	232 (122)	249 (175)	−17	—	−51 to 18
Primary postpartum haemorrhage**	6 (4)	3 (2)	—	2.00	0.50–8.32
Blood loss ≥ 500 ml**	6 (4)	2 (1.3)	—	3.00	0.61–15.53
Blood loss ≥ 1000 ml**	0	1 (0.7)	—	—	—
Repeat oxytocic injection**	13 (8.7)	10 (6.7)	—	1.30	0.56–3.13
Need of blood transfusion**	5 (3.3)	2 (1.3)	—	2.50	0.49–13.36
Manual removal of placenta**	1 (0.7)	3 (2)	—	0.33	0.03–3.20
Mean duration of third stage* (minutes)	11.6 (17.4)	10.4 (4.2)	1.2	—	−1.7 to 4.1
Duration of third stage (minutes)**					
≤10	128 (85.3)	126 (84)	—	1.02	0.59–2.08
11–30	21 (14)	23 (15.3)	—	0.91	0.47–1.71
>30	1 (0.7)	1 (0.7)	—	1	0.06–16.14

* Data are presented as mean (SD).

** Data are presented as *n* (%).

Adverse effects are presented in Table 4. The incidence of nausea (RR 0.18, 95% CI 0.04–0.78) and vomiting (RR 0.1, 95% CI 0.01–0.74) were significantly lower in the carbetocin group. The incidence of facial flushing, headache, shivering and pain over injection site were low and similar in both groups. Thirty-two percent of women in the carbetocin group had tachycardia (defined as maternal pulse ≥100) within 60 minutes postdelivery and was significantly higher than the 19% recorded in the syntometrine group (RR 1.68, 95% CI 1.03–3.57). The mean blood pressure values at different intervals after delivery of each group are shown in Table 4. The incidence rate of hypertension (defined as blood pressure ≥140/90 mmHg) at 30 and 60 minutes after delivery was 5.3 and 4% in the syntometrine group while no woman in the carbetocin group had raised blood pressure, and the difference was significant.

**Table 4:** Adverse effects profile

	Carbetocin (*n* = 150)	Syntometrine (*n* = 150)	Mean difference	RR	95% CI
Nausea*	2 (1.3)	11 (7.3)	—	0.18	0.04–0.78
Vomiting*	1 (0.7)	10 (6.7)	—	0.10	0.01–0.74
Facial flushing*	0	3 (2)	—	—	—
Headache*	1 (0.7)	2 (1.3)	—	0.50	0.05–5.54
Shivering*	2 (1.3)	6 (4)	—	0.33	0.06–1.63
Pain over injection site*	0	1 (0.7)	—	—	—
Tachycardia (pulse ≥ 100 beats per minute) within 60 minutes postdelivery*	32 (21.3)	19 (12.7)	—	1.68	1.03–3.57
Mean systolic blood pressure immediately after delivery**	113.7 (11.6)	116.7 (12.9)	−3.0	—	−5.7 to −0.2
Mean diastolic blood pressure immediately after delivery**	63.4 (9.2)	67.2 (9.4)	−3.8	—	−6.0 to −1.7
Mean systolic blood pressure 30 minutes after delivery**	112.9 (10.3)	118.1 (11.6)	−5.2	—	−7.8 to −2.8
Mean diastolic blood pressure 30 minutes after delivery**	64.5 (8.4)	68.1 (10.0)	−3.6	—	−5.7 to −1.5
Mean systolic blood pressure 60 minutes after delivery**	113.3 (10.5)	117.1 (11.9)	−3.8	—	−6.4 to −1.3
Mean diastolic blood pressure 60 minutes after delivery**	64.7 (8.7)	67.9 (8.8)	−3.2	—	−5.2 to −1.2
**Hypertension (blood pressure** ≥ 140/90 mmHg)*					
Immediately after delivery	2 (1.3)	4 (2.7)	—	0.5	0.09–2.74
30 minutes after delivery	0	8 (5.3)***	—	—	—
60 minutes after delivery	0	6 (4)****	—	—	—

* Data are presented as *n* (%).

** Data are presented as mean (SD).

*** *P* < 0.01 by Fisher‘s exact test.

**** *P* < 0.05 by Fisher’s exact test.

## Discussion

This study shows that using carbetocin as a routine uterotonic drug administered as part of the active management of the third stage in uncomplicated labour and delivery is as effective as syntometrine but with a better adverse effect profile. The comparative efficacy and its excellent tolerance are consistent with other studies where it was given after caesarean section or to women at high risk of postpartum haemorrhage.^13^–^15^ IM route is the most common and convenient route to administer uterotonic agents after vaginal delivery. Therefore, we designed this study to compare the efficacy of IM carbetocin with IM syntometrine. Our results suggest that IM carbetocin could be an alternative to IM syntometrine for the vast majority of the low-risk obstetric population.

We did not demonstrate any difference in the amount of blood loss during delivery, the incidence of primary postpartum haemorrhage and the need of additional oxytocic injection between the two groups. Despite the low incidence of postpartum haemorrhage in both treatment groups of our women, about 50 and 20% of the whole study group had more than 10 and 20% drop in haemoglobin level within 48 hours, respectively. These figures were consistent with our previous studies conducted in a similar population^7^,^16^ and confirmed that the clinical estimation of blood loss in childbirth is inaccurate.^17^,^18^ These suggested that the conventional definition of postpartum haemorrhage of blood loss > 500 ml is of little clinical use, and we agreed a peripartum fall in haemoglobin level more than 10% as the recommended diagnostic criteria for primary postpartum haemorrhage.^19^ We therefore used the haemoglobin drop as our primary outcome measure in assessing the efficacy of the uterotonic agents in reducing postpartum haemorrhage. We checked for haemoglobin change 48 hours instead of 24 hours after delivery to provide longer time for haemodynamic equilibrium. In our study, the mean fall in haemoglobin, the incidence of >10% and >20% drop in haemoglobin level, were lower in carbetocin group compared with that of the syntometrine group, but the difference was insignificant. Our data therefore suggest that IM carbetocin is as effective as syntometrine in preventing postpartum haemorrhage.

Earlier studies where 200 microgram IM carbetocin given immediately after birth found a higher incidence of retained placenta,^12^ and other subsequent studies have delayed administration until after delivery of the placenta.^15^ In our study, we administered 100 microgram carbetocin IM after the delivery of anterior shoulder but not after the placental delivery to mimic active management of the third stage of labour. Only 1 of 150 women (0.7%) in the carbetocin group required manual removal of retained placenta, and this was lower than the 2% in women in the syntometrine group, although the difference was statistically insignificant.

Carbetocin has a low incidence of adverse effects. Nausea and vomiting was recorded in 7.3 and 6.7% of women in the syntometrine group and the incidence was significantly higher than the 1.3 and 0.7% in the carbetocin group. Nausea and vomiting caused by syntometrine was also observed in our previous studies with similar incidence.^7^ Similar to oxytocin, carbetocin is less likely to cause hypertension than syntometrine. High blood pressure was recorded in 5.3 and 4% of women 30 and 60 minutes after delivery in the syntometrine group and none were recorded in the carbetocin group. Previous studies have shown that carbetocin could induce maternal tachycardia and facial flushing,^11^,^12^,^20^ and 21% of women in carbetocin group did experience tachycardia, and the incidence was significantly higher than that in the syntometrine group. Although tachycardia is usually transient and is not a serious problem, it may confound monitoring of women with bleeding where haemodynamic decompensation is often picked up through increases in heart rate before changes in the blood pressure. None of our women in the carbetocin group had facial flushing and the incidence was also low in women in the syntometrine group.

## Conclusion

IM carbetocin is as effective as IM syntometrine in preventing postpartum haemorrhage after vaginal delivery in the low-risk obstetric population. Carbetocin is less likely to induce hypertension than syntometrine. Although it is more costly, it has a low incidence of adverse effect. Carbetocin should be considered as a good alternative to conventional uterotonic agents used in managing the third stage of labour. Further studies on the use of carbetocin in women suspected or diagnosed to have hypertensive disorder or pre-eclampsia is needed to see if it could become the drug of choice for this subgroup of pregnant women.
